# Combination of highly efficient microflora to degrade paint spray exhaust gas

**DOI:** 10.1038/s41598-020-62972-2

**Published:** 2020-04-07

**Authors:** Huixia Lan, Shixin Qi, Da Yang, Heng Zhang, Jianbo Liu, Yanhui Sun

**Affiliations:** 1 College of Environment and Safety Engineering, Qingdao University of Science and Technology, Qingdao, 266042 China; 2 Fujian Provincial Key Laboratory of Ecology-Toxicological Effects & Control for Emerging Contaminants, Putian, 351100 China; 3 State Key Laboratory of Pulp and Paper Engineering, South China University of Technology, Guangzhou, 510640 China

**Keywords:** Atmospheric chemistry, Chemical engineering

## Abstract

Spray paint exhaust gas contains recalcitrant volatile organic compounds (VOCs), such as benzene, toluene and xylene (BTX). Treating BTX with a biofilter often achieves unsatisfactory results because the biofilter lacks efficient microbial community. In this work, three strains for BTX degradation were isolated and identified as *Pseudomonas putida*, *Bacillus cereus* and *Bacillus subtilis* by using 16S rRNA sequencing technology. A consortium of highly efficient microbial community was then constructed on a stable biofilm to treat BTX in a biofilter. A relatively suitable ratio of *P. putida*, *B. cereus* and *B. subtilis* was obtained. An efficiency of over 90% was achieved in the biofilter with VOC concentration of 1000 mg/m^3^ through inoculation with the microbial community after only 10 days of operation. Thus, fast start-up of the biofilter was realised. Analysis of intermediate products by gas chromatography–mass spectrometry indicated that BTX was degraded into short-chain aldehydes or acids via ring opening reactions.

## Introduction

Paint spray exhaust gas includes volatile organic compounds (VOCs), such as benzene, toluene and xylene (BTX), and even more complicated compounds, i.e. chlorinated benzenes and toluenes^1,2^, all of which can cause great harm to humans^3,4^. The indiscriminate discharge of large amounts of paint spray exhaust gas also exerts a negative impact on the atmospheric environment^5,6^. Therefore, efficient technologies should be developed to treat paint spray exhaust gas and address its effects.

Although several technologies for paint spray exhaust gas treatment exist, some of these methods are difficult to apply on a large scale because of their shortcomings^7^. Biofiltration and biotrickling filters have received wide attention because they are low-cost and occupy a small space. Moreover, the activity of microorganisms can remain comparatively stable^8–10^. Leili *et al*. found that the biofiltration system shows good performance in terms of removing BTEX^11^. In theory, VOCs can completely mineralise during biofiltration. However, it is restricted by the microorganism type and the operating parameters in practise. Thus, the effect remains unsatisfactory^12^.

The toxicants in waste gas could be degraded with a specific strain by acclimation, which can be applied to the biofilters to improve their efficiency. A removal efficiency of higher than 98% was achieved by *Bacillus firmus* when the concentration of ketone (acetone and methyl ethyl ketone) and benzene in paint spray exhaust gas was lower than 3000 mg/m^3^ ^13^. Mohammad *et al*. used *Exophiala sp*. as inoculum for biofilter, which had a good removal effect on BTEX^14^. However, because the composition of the exhaust gas might be complex, a single strain may not always obtain satisfactory results, resulting in unstable removal rates^15,16^. The microbial community has been studied to promote biofilter operation. Archaea and bacteria effectively remove printing press VOCs in an anaerobic bioscrubber^17^. Xue *et al*. used a biotrickling filter to treat complex odorous gas and found that bacteria play a greater role than fungi and actinomycetes do in degrading VOCs^18^. Lu *et al*. used *Pseudomonas*, *Kocuria*, *Arthrobacter* and *Bacillus* to treat exhaust gases containing formaldehyde (0–6.5 mg/m^3^), benzene (2.2–46.7 mg/m^3^), toluene (0.5–28.2 mg/m^3^) and xylene (4.1–59 mg/m^3^), and a maximum removal rate of 93% and stable treatment effects were obtained during continuous operation^19^. Jahangiri *et al*. and Wang *et al*. used mixed bacteria to treat a benzene series, and both groups reported excellent removal effects^20,21^. However, the removal rate is often unstable because the proportion of bacteria is not fixed^22,23^. However, few reports have been conducted on the construction of a microbial consortium that consists of the appropriate proportion of bacteria.

In this study, a high-efficiency microbial community with appropriate proportions of specific bacteria was constructed using dynamic experiments. It was then applied to a biofilter to treat the simulated spray paint exhaust gas composed of BTX. The constructed microbial community shortened the biofilm-forming time accelerated the biofilter start-up and also contributed to a stable operation, thus proving the operational flexibility of the biofilter.

## Materials and methods

### Materials

#### Simulated paint spray exhaust gas

The concentrations of BTX (benzene, toluene, and xylene) in paint exhaust gas are relatively high, and they have strong toxicity. Thus, the simulated paint waste gas mainly contained BTX. The ratio of benzene, toluene and xylene was 20:40:40.

#### Experimental installation

**Figure 1:**
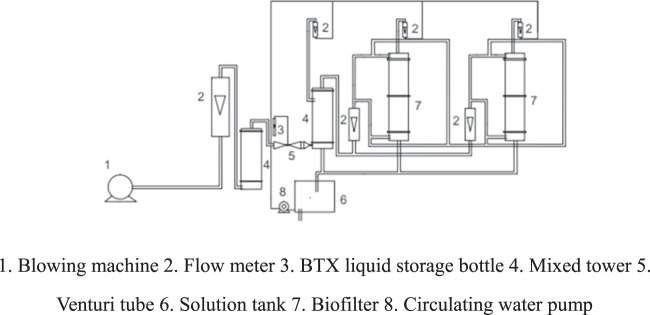
Diagram of the biofilter. 1. Blowing machine 2. Flow meter 3. BTX liquid storage bottle 4. Mixed tower 5. Venturi tube 6. Solution tank 7. Biofilter 8. Circulating water pump.

The biofilter used in the experiments is shown in Fig. 1.

The diameter of the biofilter tower was approximately 100 mm, and the bioreactor included two layers with a filling height of 250–300 mm. The filler in the packing layer was block volcanic fillers with a porosity of 0.73.

BTX evaporated from the liquid storage bottle with the assistance of the air pump and mixed with air produced by the blowing machine in a Venturi tube to obtain a certain concentration of VOCs. Then, the VOCs entered the biofilter from the bottom of the tower and were degraded by the bacteria on the packing. The purified gas discharged from the exhaust pipe at the top of the tower. Freeze-dried powders of *Pseudomonas putida*, *Bacillus cereus* and *Bacillus subtilis* were introduced into the solution tank with a dosage of 0.1 g/L and a ratio of 1:1:1, and the pH value was maintained around 7.2. The composition and concentration of the inorganic salts in the tank are shown in Table S1. The solution containing the inoculum and inorganic salts was sprayed from the top to the bottom of the biofilter via a circulating water pump connected to the solution tank.

### Analytical methods

#### rRNA sequencing

The pure culture of the strains on a plate medium was sampled and transferred to the test tube, and then the Ezup Column Bacteria Genome Extraction Kit was used to conduct DNA extraction (the detailed description is shown in Test S1). The composition of the bacteria genome extraction kit is shown in Table S2. The extracted gene fragment was amplified with the universal primers 27F (5′AGAGTT TGGATCCTGGCTCAG3′) and 1492R (5′CGGTTACCTTGTTACGACTT3′). The PCR procedure was as follows: initial denaturation for 30 s at 98 °C, and then 40 cycles at 98 °C for 10 s, 55 °C for 30 s, 72 °C for 10 s, followed by a final extension for 10 min at 72 °C. The PCR products were sequenced by Qingdao Qingke Zixi Biological Company to obtain amplified regions of 16S rRNA sequences. Nucleotide sequences were aligned with the sequence database of the National Biological Information Center.

#### VOC concentration

The simulated exhaust gas was measured using the VOC instrument of Phocheck Tiger (ION Company,). Concentrations were measured three times at the same sampling port, and the average values were reported.

#### Determination of components in the gas, liquid and biofilm phase

The components in the gas phase, liquid phase and biofilm were analysed by using gas chromatography–mass spectrometry (GC-MS). The components in the gas phase was collected by a activated carbon tube and desorbed using CS_2_ as the solvent during analysis. The components in the liquid phase were extracted using normal hexane. Water sample with a volume of 5 mL and hexane with a volume of 2 mL were added to a tube with plug and treated with ultrasound for 15 min. The upper solvent phase was taken for GC-MS analysis. The components in the biofilm were first washed with deionised water, and then the same procedure as that of the liquid phase was proceeded.

The conditions of GC-MS are described as follows: The chromatographic column was a HP-5 capillary column, and the injector temperature was 280 °C. The temperature program was as follows: the initial furnace temperature was held constant at 35 °C for 3 min and then raised to 280 °C for 4 min. The velocity of the nitrogen carrier gas was 1.8 mL/min.

## Results and Discussion

### Identification of the strains

Three strains were obtained from the activated sludge acclimated with BTX. The strains grew on the culture using BTX as the sole source of carbon and energy. The colony morphology of strain 1 was round, translucent, colourless and smooth, and it had a moist and expanded surface. The colony morphology of strain 2 had an expanded surface, ovoid in shape, dry, translucent, white and smooth. The colony morphology of strain 3 revealed a dotted distribution, swollen surface and rough boundaries. It was also dry, translucent and white.

The PCR products obtained from the three strains were sequenced. The amplified regions of the 16S rRNA sequences are shown in Fig. S1, and the matching results are shown in Tables S2, S3 and S4. The matching index of strain 1 with *P. putida*, *P. putida* OKF01, and *P. putida* S50 was 99%. Thus, strain 1 was identified as *P. putida*. The matching index of strain 2 with *B. cereus* strain SIIA-Pb-E3, *B. cereus* strain ATCC 14579 and *B. cereus* strain LH8 was 99%. Therefore, strain 2 was identified as *B. cereus*. The matching index of strain 3 with *B. subtilis* strain G-13, *B. subtilis* strain CR26 and *B. subtilis* strain FY99 was 99%. Therefore, strain 3 was identified as *B. subtilis*.

### Dynamic construction of specified microbial community

**Figure 2:**
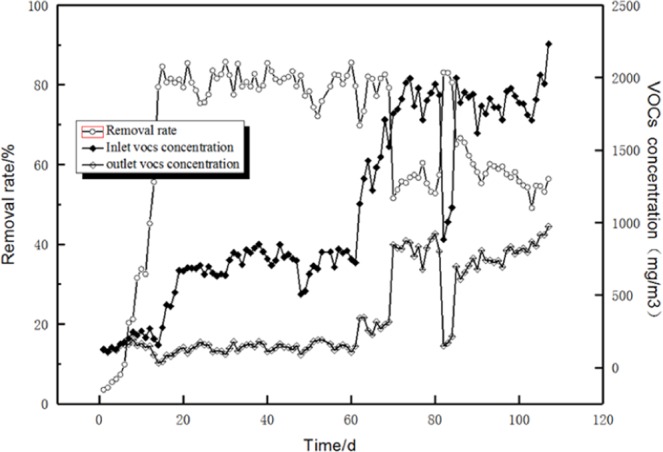
Effect of operating time on the removal efficiency of the biofilter.

The removal rate of VOCs by the specific bacteria is an important parameter of biofilters^24,25^. The biofilter was filled with volcanic rocks, and the gas flow rate was controlled between 0.5 and 2.5 m^3^/h. The circulating solution rate was controlled to 0.5–2.5 L/h, and the empty bed retention time (EBRT) was set to 30 s. The biofilter was initially operated at a low VOC concentration of 100–300 mg/m^3^. Thereafter, the concentration of inlet VOCs increased gradually with time and reached 2000 mg/m^3^ on day 70. Figure 2 shows the removal efficiency of the biofilter with time.

Figure 2 shows that the outlet concentration of VOCs was slightly lower than the inlet concentration at the initial stage of operation for a low removal rate of the biofilter. A biofilm on the filler did not form at this stage. Then, the removal rate of VOCs increased with time. After 15 days of continuous operation, the treatment efficiency was higher than 80%, and a biofilm began to form on the surface of the filler. The bacteria on the surface of the fillers began to use benzene, toluene and xylene in the exhaust gas as carbon and energy sources.

The biofilm distribution on the surface of volcanic rock filler was uniform, and the colour was fawn. Under the same EBRT conditions and circulating liquid flow rate, it achieved a stable operation state earlier than that reported by Vijayan *et al*.^26^. This result might be due to the use of the screened strains as inoculum, which could shorten the biofilm formation time.

The removal efficiency of the biofilter exceeded 80% when the inlet gas concentrations were in the range of 150–1500 mg/m^3^. Chen *et al*. used microbes bound to the wheat bran/red wood powder/diatomaceous earth carrier as inoculants to a biotrickling filter for treating BTo-X, and they found that the removal rate of BTo-X decreased with the increase in inlet loading^27^. In this study, we obtained the same trend. When the exhaust gas concentration increased to approximately 2000 mg/m^3^ (inlet load, 250 g·m^−3^·h^−1^), the removal rate obviously decreased, which might be due to the toxicity of the high concentration of the BTX to the bacteria and the inhibition of the activity of the bacteria in the biofilm. Thus, the bacteria could not effectively degrade high concentrations of exhaust gas. The toxicity of TVOCs in this study was stronger than that of toluene alone because of the presence of benzene.

### Construction of a highly efficient microbial consortium

The results of the dynamic experiments indicate that a stable and efficient biofilm was formed on the packing surface. When the biofilter was filled with volcanic rock, the EBRT was 30 s, the organic load was approximately 250 g·m^−3^·h^−1^, the TVOC concentration was approximately 1000 mg·m^−3^ and the removal rate of the filter approximately 90% after 50 days of operation. The biofilm on the surface of the filter was picked and mixed with deionised water, the mixed suspension was diluted with deionised water by gradient dilution and then colony counting was carried out by colony counting technique.

The colony counting results showed that the number of *P. putida*, *B. cereus* and *B. subtilis* in the 10^–4^ dilution ratio was 68, 88 and 244 with triple average, respectively. Thus, the ratio of *P. putida*, *B. cereus* and *B. subtilis* was 17:22:61.

### Treatment effect of the high-efficiency microbial community

**Figure 3:**
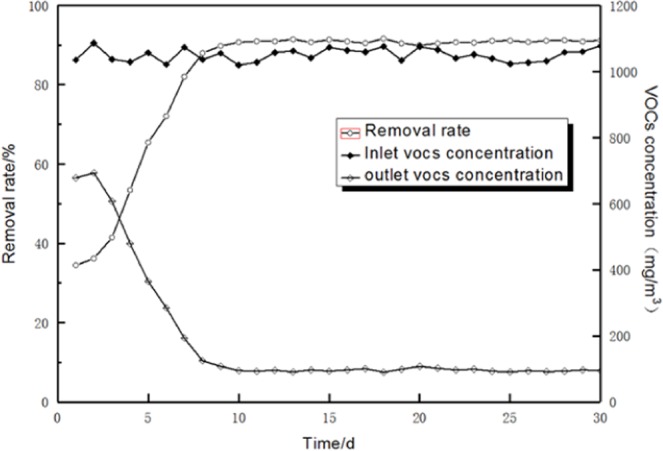
Removal of VOCs by the high-efficiency microbial combination.

A microbial consortium composed of *P. putida*, *B. cereus* and *B. subtilis* at a ratio of 17:22:61 was prepared in this study. The biological trickling tower was filled with volcanic rock, the EBRT was set to 30 s, the inlet simulated exhaust gas concentration was maintained at approximately 1000 mg/m^3^ and the organic load was set to approximately 250 g/(m^3^.h). Changes in the inlet-simulated exhaust gas concentration, outlet concentration and removal rate with time are shown in Fig. 3.

Figure 3 revealed that the outlet concentration decreased rapidly with time and stabilised on day 9. The removal rate reached 89.8% and the outlet concentration of the biofilter was 108 mg/m^3^. After 10 days, the biofilter showed a stable operation, and its removal rate exceeded 90%. The removal rates of benzene, toluene and xylene were over 86%, 91% and 92%, respectively. Moreover, the biofilm-forming time considerably shortened, and the processing capacity of the biofilter enhanced. Hu *et al*. used a combination of *Zoogloea resiniphila* HJ1 and *Methylobacterium rhodesianum* H13 as an inoculum to start biotrickling filters for toluene, *o*-xylene and dichloromethane removal, a fast start-up and more than 96.5% removal efficiency achieved on day 17^28^. Zhou *et al*. used *Ralstonia pickettii* L2 and sludge as inoculum in the biotrickling filters and achieved fast start-up and more than 90% chlorobenzene removal on day 14^29^. Using a combination of specific strains as inoculum is beneficial to the start-up of biotrickling filters. In this study, the biofilter was inoculated with the appropriate ratio of *P. putida*, *B. cereus* and *B. subtilis*. The TVOC removal efficiency exceeded 90% on day 10, indicating a faster start-up than those achieved by Hu *et al*. and Zhou *et al. P. putida*, *B. cereus* and *B. subtilis* were able to metabolise BTX as sole carbon and energy source^30,31^. The laccase contained in the *P. putida* can oxidise the benzene ring to produce phenols or cyclohexenol^32,33^. *B. cereus* has a high ability to degrade toluene^34^, while *B. subtilis* has a better ability to degrade benzene than toluene and xylene^35^ and easily causes the ring opening reaction of benzene ring^36^. Thus, the three bacteria could form a co-metabolic relationship. Studies reported that the degradation of BTX by a combination of *P. putida*, *B. cereus* and *B. subtilis* was superior to that by a single bacterium^34,35,37^, which was consistent with the results of this study. In recent years, fungi have been reported to degrade BTX effectively. Prenafeta-Boldú *et al*. found that fungi had high efficiencies in the degradation of BTX^38^. However, fungi will be more abundant in biofilters operated at a low water content. In this study, spray was applied directly to the packing, thus resulting in a rather humidity. As a result, almost no fungi were present.

The compositions of pollutants in the gas phase, liquid phase and biofilm were analysed by GC-MS on day 20 to investigate the degradation pathway of VOCs by the microbial consortium. The components in the gas phase are shown in Table 1.

**Table 1:** Main components of the gas phase on day 20.

Peak number	Retention time	Peak area	Material name	Basic molecular structure
1	4.681	3.23 × 10^8^	benzene	
2	5.012	3.35 × 10^8^	*o*-xylene	
3	5.391	3.01 × 10^8^	methyl benzene	
4	5.405	22,723	*o*-dihydroxybenzene	
5	5.514	16,408	3-hexanol	
6	6.215	8229	benzyl alcohol	

Table 1 reveals that the gas phase contains BTX, catechol, 3-hexanol and benzyl alcohol. Under aerobic conditions, benzenes initially transform into intermediate products of easy-to-open rings under the action of oxygenase. Further orthocleavage of the ring occurred, and straight-chain organic compounds, including aldehydes and acids, formed^39^. These organics are eventually mineralised completely through the tricarboxylic acid cycle. Catechol and benzyl alcohol in the gas phase were transformed into intermediate products before BTX ring opening, and the peak area of *o*-diphenol was relatively large. Thus, the benzene series was transformed into intermediate, in which ring opening occurred comparatively easily. Few intermediate products were detected in the gas phase, and their concentrations were relatively low. The products of the degradation of the bacteria might be soluble in water, and most of them are abundant in the liquid phase and biofilm. Only volatile intermediates were detected in the gas phase. Some studies indicated that *P. putida* has two main steps in the degradation of BTX. Firstly, methyl or ethyl substituents of the benzene ring were broken down by monooxygenase and subsequently transformed by several oxidations to the corresponding substituted pyrocatechols^40^. The following mineralisation of those intermediaries to produce CO_2_ and H_2_O occurred^41^. In this study, catechol was detected, confirming this degradation pathway of BTX.

The compositions of pollutants in the liquid phase and biofilm on day 20 are shown in Tables 2 and 3.

**Table 2:** Main components in the liquid phase on day 20.

Peak number	Retention time	Peak area	Material name	Const Basic molecular structure itutional formula
1	3.905	12,723	aldehyde	
2	5.323	45,312	methylbenzene	
3	5.489	121,240	2-hexanol	
4	6.834	19,435	*o*-dihydroxybenze	
5	7.014	43,291	benzaldehyde	
6	7.232	86,123	benzoic acid	
7	9.981	24,215	adipic acid

**Table 3:** Main components in the biofilm at 20th days.

Peak number	Retention time	Peak area	Material name	Basic molecular structure
1	5.505	95,911	*o*-dihydroxybenzene	
2	6.324	36,408	benzyl alcohol	
3	6.851	38,229	2-hexanol	
4	9.961	21,245	adipic acid	

The main components in the liquid phase contained acetaldehyde, catechol, toluene, 2-hexanol, benzaldehyde, benzoic acid and adipic acid. Table 3 shows that catechol, benzyl alcohol, 2-hexanol and adipic acid were contained in the biofilm. The components in the biofilm were in line with those in the liquid phase, mainly because the compounds were hydrophilic and could enter the liquid phase through the biofilm. The peak areas of catechol and benzyl alcohol in the liquid and biofilm phases were larger than those in the gas phase. Dou *et al*. found that benzoate was the intermediate of benzene oxidation by strain *B. cereus*. The benzene was probably metabolised by an initial hydroxylation to form phenol, which was then converted to benzoate^42^. Benzoic acid was also detected in the liquid phase in this study, in accordance with the findings of Dou *et al*.

A small amount of acetaldehyde was detected in the liquid phase. The presence of acetaldehyde is formed during metacleavage of toluene or xylene through the conversion of 4-hydroxy-2-oxo-valerate to acetaldehyde and pyruvate. 2-hexanol and adipic acid are easily mineralised via the tricarboxylic acid cycle under the action of dehydrogenases^43^. Therefore, the peaks of these intermediates in biofilm were low.

With toluene taken as an example, the degradation process by the microbial consortium occurred as follows: benzyl alcohol, benzaldehyde, cis-1, 6-dihydroxy-2, 4-cyclohexadiene-1-carboxylic acid and catechol are formed by the action of an initial monooxygenase in the bacteria. The intermediate products are then mineralised into carbon dioxide and water through the tricarboxylic acid cycle under the action of dehydrogenase.

## Conclusions

In this work, three strains were isolated and identified as *P. putida*, *B. cereus* and *B. subtilis*. The bacteria were then used to degrade main recalcitrant VOCs (benzene, toluene and xylene) in spray paint exhaust gas. *P. putida*, *B. cereus* and *B. subtilis* were combined with a relatively suitable ratio and inoculated to a biofilter. The VOC degradation efficiency of the biofilter exceeded 90% after 10 days of operation. BTX were degraded into short-chain aldehydes or acids via ring opening reactions and finally mineralised to CO_2_ and H_2_O by the microbial consortium. The biofilter inoculated with the microbial consortium achieved fast start-up and high efficiency in BTX degradation.

## Supplementary information

Supporting Information.
