# Supplementary material for: Combination of highly efficient microflora to degrade paint spray exhaust gas
Source: Sci Rep. 2020 Apr 7;10:6027. doi: 10.1038/s41598-020-62972-2 (PMC7138788; doi:10.1038/s41598-020-62972-2)
Supplement: Supplementary file 1 — Supporting Information. [file 41598_2020_62972_MOESM1_ESM.docx]

**Supporting Information**

**Combination of highly efficient microflora to degrade paint spray exhaust gas**

Huixia Lan^1,2,*^ , Shixin Qi^1^, Da Yang^1^, Heng Zhang^1^, Jianbo Liu^1^, Yanhui Sun^1,3^

**Tab.S1 Composition and concentration of inorganic salts in spray liquid**

| Composition | concentration(g/L^-1^) | Composition | concentration (g/L^-1^) |
| --- | --- | --- | --- |
| NaNO_3_ | 0.01 | MnSO_4_·H_2_O | 0.042 |
| Na_2_HPO_4_  KH_2_PO_4_  CoCl_2_·6H_2_O | 0.7  0.52  0.0077 | CuCl_2_·2H_2_O  CaCl_2_·2H_2_O  Na_2_MoO_4_ | 0.00 6  0.054  0.0009 |
| FeSO_4_(NH_4_)_2_SO_4_·6H_2_O  ZnSO_4_·7H_2_O | 0.0102  0.0009 | NH_4_Cl  CaCl_2_ | 0.6  0.04 |

**Test S1. The steps of DNA extraction**

The composition of bacteria genome extraction Kit was shown in Tab.S2.

(1) The selected strains were expanded and cultured at 37℃ to obtain bacteria, add in 180 µL Buffer Digestion, and add 20 µL Proteinase K solution, blending oscillation. Water bath at 56℃ for 1h until the cells are completely lysed. Add in 180 µL Buffer Digestion, and add 20 µL Proteinase K solution, blending oscillation. Water bath at 56℃ for 1h until the cells are completely lysed.

(2)The strain was placed in a centrifuge tube sterilized at high temperature, in which 180µL lysozyme solution was precisely added with a pipette, After that, it was placed in water bath at a temperature of 37℃, and 20µL Proteinase K solution was added after 45min. The centrifugal tube was oscillated by an oscillator and heated in a water bath at 56℃ for 1h.

(3) In the centrifuge tube obtained in the previous step, precisely remove 200µL of Buffer BD liquid, and fully invert and mix.

(4)Add 200µL of anhydrous ethanol and mix thoroughly.

(5) The adsorption column was placed in the collecting tube, and the solution and translucent fibrous suspended matter were precisely removed to the adsorption column with a high-temperature sterilized pipette. After standing for 2min, the column was centrifugated at a speed of 12000rpm for 1min to preserve the adsorption column.

(6) Put the adsorption column back into the collection tube, add 500μL PW Solution, centrifuge for 30s at 10000rpm, and pour out the filtrate.

(7) Put the adsorption column back into the collection tube, add 500μL Solution of Wash, centrifuge for 30s at 10000rpm.

(8)The adsorption column was put back into the collecting tube, centrifuged at 12000rpm for 1min.

(9) The adsorption column was removed and put into a new 1.5ml centrifuge tube. The CE Buffer solution of 50~ 100µL was precisely removed and centrifuged at 12000rpm for 2min after 3min to collect the DNA solution. The extracted DNA can be immediately processed for further experiments or stored at -2℃.

The

**Tab.S2 Bacterial Genome Extraction Kit**

| Composition | B518225-0050 | B518225-0100 |
| --- | --- | --- |
| Buffer Digestion | 10mL | 20mL |
| Buffer BD | 12mL | 24mL |
| PW Solution(concentrate) | 18mL | 36mL |
| Wash Solution(concentrate) | 7.5mL | 15mL |
| CE Buffer(pH 9.0) | 15mL | 30mL |
| Proteinase K | 1.2mL | 2.4mL |
| Enzymatic lysis buffer | 10mL | 20mL |

**Tab.S3 The 16S rRNA sequence alignment results of Colony 1**

| Accession | comparison | Ident |
| --- | --- | --- |
| [KP795886.1](https://www.ncbi.nlm.nih.gov/nucleotide/803471487?report=genbank&log$=nucltop&blast_rank=1&RID=7T6E3XEM014) | [Pseudomonas putida. 16S ribosomal RNA gene, partial sequence](https://blast.ncbi.nlm.nih.gov/Blast.cgi#alnHdr_803471487) | 99% |
| [KC969074.1](https://www.ncbi.nlm.nih.gov/nucleotide/572168581?report=genbank&log$=nucltop&blast_rank=2&RID=7T6E3XEM014) | [Pseudomonas putida OKF01 16S ribosomal RNA gene, partial sequence](https://blast.ncbi.nlm.nih.gov/Blast.cgi#alnHdr_572168581) | 99% |
| [KC466255.1](https://www.ncbi.nlm.nih.gov/nucleotide/498541719?report=genbank&log$=nucltop&blast_rank=3&RID=7T6E3XEM014) | [Pseudomonas putida. S50 16S ribosomal RNA gene, partial sequence](https://blast.ncbi.nlm.nih.gov/Blast.cgi#alnHdr_498541719) | 99% |

**Tab.S4 The 16S rRNA sequence alignment results of Colony 2**

| Accession | comparison | Ident |
| --- | --- | --- |
| [KX036611.1](https://www.ncbi.nlm.nih.gov/nucleotide/1026605014?report=genbank&log$=nucltop&blast_rank=1&RID=7T788047014) | [Bacillus cereus strain SIIA_Pb_E3 16S ribosomal RNA gene, partial sequence](https://blast.ncbi.nlm.nih.gov/Blast.cgi#alnHdr_1026605014) | 99% |
| [KF863832.1](https://www.ncbi.nlm.nih.gov/nucleotide/582987478?report=genbank&log$=nucltop&blast_rank=2&RID=7T788047014) | [Bacillus cereus strain ATCC 14579 16S ribosomal RNA gene, partial sequence](https://blast.ncbi.nlm.nih.gov/Blast.cgi#alnHdr_1026605014) | 99% |
| [KC248215.1](https://www.ncbi.nlm.nih.gov/nucleotide/442539368?report=genbank&log$=nucltop&blast_rank=3&RID=7T788047014) | [Bacillus cereus strain ATCC 14579 16S ribosomal RNA gene, partial sequence](https://blast.ncbi.nlm.nih.gov/Blast.cgi#alnHdr_1026605014) | 99% |

**Tab.S5 The 16S rRNA sequence alignment results of Colony 3**

| Accession | comparison | Ident |
| --- | --- | --- |
| [KJ139434.1](https://www.ncbi.nlm.nih.gov/nucleotide/601093280?report=genbank&log$=nucltop&blast_rank=1&RID=7T73U2DD015) | [Bacillus subtilis strain G-13 16S ribosomal RNA gene, partial sequence](https://blast.ncbi.nlm.nih.gov/Blast.cgi#alnHdr_601093280) | 99% |
| [KR780430.1](https://www.ncbi.nlm.nih.gov/nucleotide/939467363?report=genbank&log$=nucltop&blast_rank=2&RID=7T73U2DD015) | [Bacillus subtilis strain CR26 16S ribosomal RNA gene, partial sequence](https://blast.ncbi.nlm.nih.gov/Blast.cgi#alnHdr_939467363) | 99% |
| [KR029823.1](https://www.ncbi.nlm.nih.gov/nucleotide/913161264?report=genbank&log$=nucltop&blast_rank=3&RID=7T73U2DD015) | [Bacillus subtilis strain FY99 16S ribosomal RNA gene, partial sequence](https://blast.ncbi.nlm.nih.gov/Blast.cgi#alnHdr_601093280) | 99% |


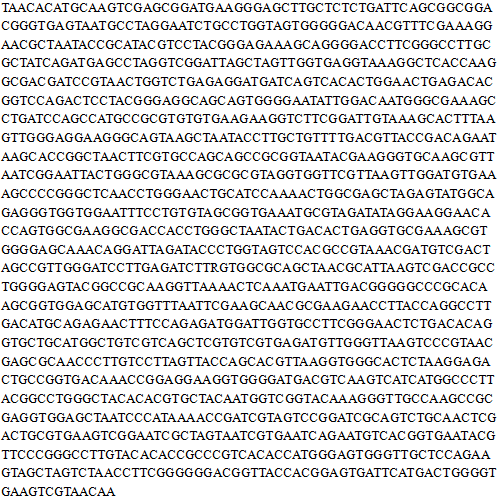

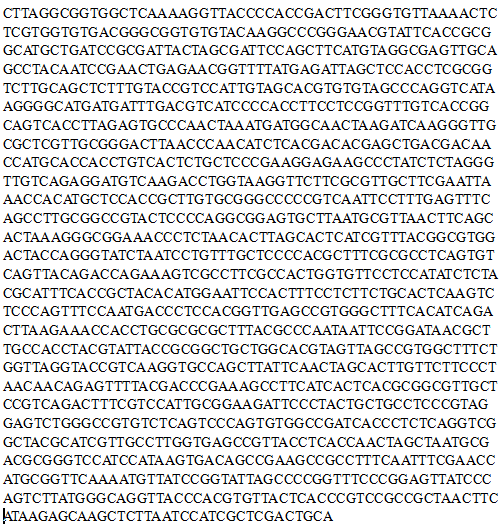


**(a) Strain 1 (b) Strain 2**


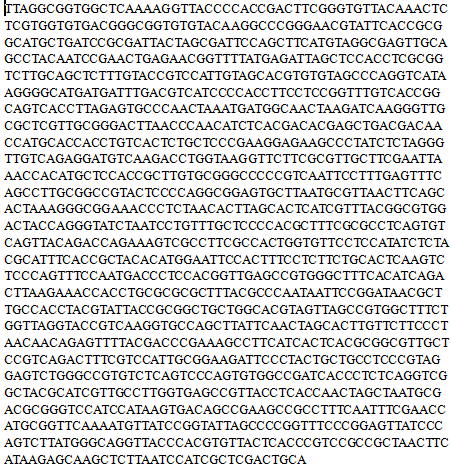


**(c) Strain 3**

**Figure S1 Amplified regions of the 16S rRNA sequences of the strains**
